# Older Adults Report Greater Limitations in Hand Dexterity Versus Whole Arm Strength Tasks

**DOI:** 10.1093/geroni/igaf122.2870

**Published:** 2025-12-31

**Authors:** Qintong Bao, Rachel Logue Cook, Susan Brown

**Affiliations:** University of Michigan, Ann Arbor, Michigan, United States; University of Michigan, Ann Arbor, Michigan, United States; University of Michigan, Ann Arbor, Michigan, United States

## Abstract

Upper extremity function, essential for most daily activities, declines with age. Such declines have been documented using validated survey instruments but to what extent these age-related changes reflect deficits in, for example, strength versus coordinated hand dexterity is unclear. Therefore, this study assessed age differences in self-reported dexterity versus strength limitations in older adults using national health survey data. Response data from 337 males and 462 females aged 60-96 years were obtained from the 2020 Health and Retirement Study wave, which included questions focused on upper extremity difficulties. We categorized these questions into two groups based on whether the activity relied predominantly on strength or hand dexterity. Group limitation scores were compared across age and gender. When adjusted for gender, older adults reported greater dexterity limitations compared to activities more dependent upon strength (p = 0.01). For dexterity-based activities and compared to male respondents, females reported more limitations which increased with age (β = 0.038, 95% CI: 0.012–0.066, p = 0.005). No significant interaction effects of age or gender on strength scores were observed (p = 0.34). Older adults reported greater limitations in tasks requiring dextrous hand control compared to actions involving whole-arm strength. Further, greater dexterity limitations increased with age for females only while strength limitations were relatively stable regardless of age. These findings highlight the importance of monitoring hand dexterity in addition to upper extremity muscle strength to maintain functional independence.

